# High Dimensional Atomic States of Hydrogenic Type: Heisenberg-like and Entropic Uncertainty Measures

**DOI:** 10.3390/e23101339

**Published:** 2021-10-14

**Authors:** Jesús S. Dehesa

**Affiliations:** 1 Departamento de Física Atómica, Molecular y Nuclear, Universidad de Granada, 18071 Granada, Spain; dehesa@ugr.es; 2 Instituto Carlos I de Física Teórica y Computacional, Universidad de Granada, 18071 Granada, Spain

**Keywords:** high dimensional hydrogenic systems, Rényi entropies, Shannon entropies, Heisenberg-like uncertainty measures

## Abstract

High dimensional atomic states play a relevant role in a broad range of quantum fields, ranging from atomic and molecular physics to quantum technologies. The *D*-dimensional hydrogenic system (i.e., a negatively-charged particle moving around a positively charged core under a Coulomb-like potential) is the main prototype of the physics of multidimensional quantum systems. In this work, we review the leading terms of the Heisenberg-like (radial expectation values) and entropy-like (Rényi, Shannon) uncertainty measures of this system at the limit of high *D*. They are given in a simple compact way in terms of the space dimensionality, the Coulomb strength and the state’s hyperquantum numbers. The associated multidimensional position–momentum uncertainty relations are also revised and compared with those of other relevant systems.

## 1. Introduction

High dimensional quantum states (HDQS), often referred to as *pseudoclassical* states, play a fundamental and practical role in numerous scientific and technological areas such as atomic and molecular chemistry and physics [1,2,3,4,5,6,7,8], quantum information and computation [9,10,11], quantum cosmology [12] and quantum technologies [13,14,15,16]. It has been observed that physics is much simpler when the space dimensionality *D* is very high in numerous quantum systems from single-particle systems subject to a central potential to more complex systems and phenomena such as, for example, random walks, quantum liquids and some quantum field models containing SU(D) gauge fields [7,17,18,19,20]. Indeed, the high dimensional (D→∞) limit is the starting point of a very useful strategy developed by Dudley R. Herschbach et al. [4,21,22,23] in atoms and molecules: the *D*-dimensional scaling method. This method needs to solve a finite many-electron problem in the (high *D*)-limit and then, perturbation theory in 1/D is used to have an approximate result for the standard dimension (D=3), obtaining at times a quantitative accuracy comparable to the self-consistent Hartree–Fock calculations. The electrons in the (high *D*)-limit of a many-electron system assume fixed positions relative to the nuclei and each other, in the D-scaled space [3].

The uncertainty measures of the Heisenberg (radial expectation values, variance) and entropy (Shannon, Rényi) types, which quantify the spreading properties of the electronic probability density, have recently been determined [24,25,26,27] for the *D*-dimensional hydrogenic system at all *D* from first principles; that is, in terms of the dimensionality *D*, the strength of the Coulomb potential (the nuclear charge) and the *D* hyperquantum numbers $\left(\eta ,\mu_{1},\mu_{2},\ldots ,\mu_{D-1}\right)$, which characterize the quantum state under consideration. This has been possible because the physical solutions (wavefunctions) of the corresponding Schrödinger equation are expressed by means of the Laguerre and Gegenbauer polynomials, which have a great deal of simple and useful algebraic properties.

However, the theoretical expressions found for the uncertainty measures of general *D*-dimensional hydrogenic states [24,25,26,27] provide with algorithmic procedures to find their numerical values, but they are somewhat highbrow and not so handy for analytical manipulations. This is because they require the numerical evaluation at the unity of a generalized univariate hypergeometric functions of ${}_{p+1}F_{p}\left(z\right)$ type (Heisenberg-like measures), a multivariate Lauricella function of type A of *s* variables and 2s+1 parameters $F_{A}^{\left(s\right)}\left(x_{1},\ldots ,x_{s}\right)$ [28,29], and a *r*-variate Srivastava–Daoust function $F_{1:1;\ldots ;1}^{1:2;\ldots ;2}\left(x_{1},\ldots ,x_{r}\right)$ [30,31] (Shannon and Rényi entropies).

In this work, we briefly review and show, in a simple, transparent and compact form, the Heisenberg measures and the Shannon and Rényi entropies of the high-dimensional (D→∞) hydrogenic states by use of some recent mathematical tools [32,33,34,35] relative to the asymptotics (α→∞) of the underlying integral functionals of Laguerre polynomials $L_{k}^{\left(\alpha \right)}\left(x\right)$ and Gegenbauer polynomials $C_{k}^{\left(\alpha \right)}\left(x\right)$, which control the hydrogenic wavefunctions as already said, the polynomial parameter α being a linear function of the space dimensionality.

The structure of this work is as follows. In Section 2, the wave functions for the multidimensional hydrogenic states in both position and momentum spaces are briefly described, and the associated probability densities are shown. Later, in Section 3 and Section 4, the uncertainty measures of Heisenberg, Rényi and Shannon types are shown and reviewed for the high dimensional hydrogenic states, respectively, in the two conjugated spaces. Finally, some conclusions are given.

## 2. Position and Momentum Probability Densities of Multidimensional Hydrogenic States

In this section, we briefly describe the stationary wavefunctions for the bound states of the D-dimensional hydrogenic system (i.e., a negatively-charged particle moving around a positively charged core under the Coulomb potential $V_{D}\left(r\right)=-\frac{Z}{r}$, being $r=|\vec{r}|,D\;⩾\;2$, and *Z* the charge of the nuclear core, assumed to be pointwise and located at the origin) and the associated electron probability densities in both position and momentum spaces. Atomic units are used from here onwards. In position space, the time-independent Schrödinger equation of this system is:

(1) $$\left(-\frac{1}{2}{\vec{\nabla }}_{D}^{2}-\frac{Z}{r}\right)\Psi (\vec{r})=E\Psi (\vec{r}),$$

where ${\vec{\nabla }}_{D}$ denotes the *D*-dimensional gradient operator, *Z* is the nuclear charge, and the electronic position vector $\vec{r}=\left(x_{1},\ldots ,x_{D}\right)$ in hyperspherical units is given as $\left(r,\theta_{1},\theta_{2},\ldots ,\theta_{D-1}\right)\equiv \left(r,\Omega_{D-1}\right)$, $\Omega_{D-1}\in S^{D-1}$, where $r\equiv |\vec{r}|=\sqrt{\sum_{i=1}^{D}x_{i}^{2}}\in \left[0\;;\;+\infty \right)$ and $x_{i}=r\left(\prod_{k=1}^{i-1}\sin\theta_{k}\right)\cos\theta_{i}$ for 1≤i≤D and with $\theta_{i}\in \left[0\;;\;\pi \right),i<D-1$, $\theta_{D-1}\equiv \phi \in \left[0\;;\;2\pi \right)$. The physical eigensolutions of this equation are known [36,37,38,39] to be given by the energies

(2) $$E=-\frac{Z^{2}}{2\eta^{2}},\;\;\eta =n+\frac{D-3}{2};\;n=1,2,3,\ldots ,$$

(where *n* is the principal hyperquantum number associated to the radial variable *r*) and the associated eigenfunctions:

(3) $$\Psi_{n,l,\left\{\mu \right\}}\left(\vec{r}\right)=R_{n,l}\left(r\right)\times Y_{l,\{\mu \}}\left(\Omega_{D-1}\right),$$

where $\left(l,\left\{\mu \right\}\right)\equiv \left(l\equiv \mu_{1},\mu_{2},\ldots ,\mu_{D-1}\right)$ denote the hyperquantum numbers associated to the angular variables $\Omega_{d-1}\equiv \left(\theta_{1},\theta_{2},\ldots ,\theta_{D-1}\right)$, which may take all values consistent with the inequalities $l\equiv \mu_{1}\geq \mu_{2}\geq \ldots \geq \left|\mu_{D-1}\right|\equiv \left|m\right|\geq 0$. The angular parts of the eigenfunctions are the hyperspherical harmonics, $Y_{l,\{\mu \}}\left(\Omega_{D-1}\right)$, defined [36,40,41,42] as

(4) $$Y_{l,\{\mu \}}\left(\Omega_{D-1}\right)=\frac{1}{\sqrt{2\pi }}e^{im\phi }\prod_{j=1}^{D-2}{\tilde{C}}_{\mu_{j}-\mu_{j+1}}^{\left(\alpha_{j}+\mu_{j+1}\right)}\left(\cos\theta_{j}\right){\left(\sin\theta_{j}\right)}^{\mu_{j+1}},$$

where $2\alpha_{j}=D-j-1$, ${\tilde{C}}_{m}^{\left(\alpha \right)}\left(t\right)$ denotes the orthonormal Gegenbauer or ultraspherical polynomial [28] of degree *m* and parameter α, and with the values $0\leq \theta_{j}\leq \pi$ (j=1,2,⋯,D−2) and 0≤ϕ≤2π. These hyperfunctions satisfy the orthonomalization condition:

(5) $$\int_{S_{D-1}}Y_{l^{\prime },\left\{\mu^{\prime }\right\}}^{*}\left(\Omega_{D-1}\right)Y_{l,\left\{\mu \right\}}\left(\Omega_{D-1}\right)d\Omega_{D-1}=\delta_{l,l^{\prime }}\delta_{\left\{\mu \right\}\left\{\mu^{\prime }\right\}}.$$

The symbol $R_{n,l}\left(r\right)$ of Equation (3) denote the radial part of the position wavefunction known to be given as:

(6) $$\begin{array}{cc} R_{n,l}\left(r\right) & =K_{n,l}{\left(\frac{r}{\lambda }\right)}^{l}e^{-\frac{r}{2\lambda }}L_{n-l-1}^{\left(2l+D-2\right)}\left(\frac{r}{\lambda }\right)\\ \; & =K_{n,l}{\left[\frac{\omega_{2L+1}\left(\tilde{r}\right)}{{\tilde{r}}^{D-2}}\right]}^{1/2}L_{\eta -L-1}^{2L+1}\left(\tilde{r}\right)\\ \; & ={\left(\frac{\lambda^{-D}}{2\eta }\right)}^{1/2}{\left[\frac{\omega_{2L+1}\left(\tilde{r}\right)}{{\tilde{r}}^{D-2}}\right]}^{1/2}{\tilde{L}}_{\eta -L-1}^{2L+1}\left(\tilde{r}\right), \end{array}$$

where

(7) $$\begin{array}{cc} L & =l+\frac{D-3}{2},\;l=0,1,2,\ldots \end{array}$$

(8) $$\begin{array}{cc} \tilde{r} & =\frac{r}{\lambda },\;\;\lambda =\frac{\eta }{2Z} \end{array}$$

denote the “grand orbital angular momentum quantum number” *L* and the adimensional parameters $\left(\tilde{r},\lambda \right)$, respectively. In addition, $\omega_{s}\left(x\right)=x^{s}e^{-x},\;s=2l+D-2,$ is the weight function of the Laguerre polynomials with parameter *s*. The symbols $L_{n}^{s}\left(x\right)$ and ${\tilde{L}}_{n}^{s}\left(x\right)$ denote the orthogonal and orthonormal, respectively, Laguerre polynomials with respect to the weight $\omega_{s}\left(x\right)=x^{s}e^{-x}$ on the interval $\left[0,\infty \right)$, so that:

(9) $${\tilde{L}}_{m}^{s}\left(x\right)={\left(\frac{m!}{\Gamma (m+s+1)}\right)}^{1/2}L_{m}^{s}\left(x\right),$$

and finally,

(10) $$K_{n,L}=\lambda^{-\frac{D}{2}}{\left\{\frac{(\eta -L-1)!}{2\eta (\eta +L)!}\right\}}^{\frac{1}{2}}={\left\{{\left(\frac{2Z}{n+\frac{D-3}{2}}\right)}^{D}\frac{(n-l-1)!}{2\left(n+\frac{D-3}{2}\right)\left(n+l+D-3\right)!}\right\}}^{\frac{1}{2}}\equiv K_{n,l}$$

represents the normalization constant, which ensures that $\int {\left|\Psi_{\eta ,l,\left\{\mu \right\}}\left(\vec{r}\right)\right|}^{2}d\vec{r}=1$.

In momentum space, the eigenfunctions for a generic stationary state (n,l,{μ}) of the *D*-dimensional hydrogenic system can be obtained via the Fourier transform of the position eigenfunctions (3),

(11) $${\tilde{\Psi }}_{n,l,\{\mu \}}\left(\vec{p}\right)=\int_{R_{D}}e^{-i\vec{p}\cdot \vec{r}}\;\Psi_{n,l,\left\{\mu \right\}}\left(\vec{r}\right)\;d^{D}\vec{r},$$

obtaining

(12) $${\tilde{\Psi }}_{n,l,\{\mu \}}\left(\vec{p}\right)=M_{n,l}\left(p\right)\times Y_{l,\{\mu \}}\left(\Omega_{D-1}\right),$$

where $\vec{p}=\left(p,\theta_{1},\ldots ,\theta_{D-1}\right)$, and the radial momentum wavefunction is:

(13) $$\begin{array}{ccc} M_{n,l}\left(p\right) & = & K_{n,l}^{\prime }\frac{{\left(\eta \tilde{p}\right)}^{l}}{{\left(1+\eta^{2}{\tilde{p}}^{2}\right)}^{L+2}}\;\;C_{\eta -L-1}^{\left(L+1\right)}\left(\frac{1-\eta^{2}{\tilde{p}}^{2}}{1+\eta^{2}{\tilde{p}}^{2}}\right)\\ & = & {\left(\frac{\eta }{Z}\right)}^{D/2}{\left(1+y\right)}^{3/2}{\left(\frac{1+y}{1-y}\right)}^{\frac{D-2}{4}}\sqrt{\omega_{L+1}^{*}\left(y\right)}\;\;{\tilde{C}}_{\eta -L-1}^{L+1}\left(y\right), \end{array}$$

with $y=\frac{1-\eta^{2}{\tilde{p}}^{2}}{1-\eta^{2}{\tilde{p}}^{2}}$, the dimensionless parameter $\tilde{p}=\frac{p}{Z}$ and the normalization constant:

(14) $$\begin{array}{ccc} K_{n,l}^{\prime } & = & 2^{2L+3}{\left(\frac{(\eta -L-1)!}{2\pi (\eta +L)!}\right)}^{1/2}\Gamma \left(L+1\right)\eta^{\frac{D+1}{2}}\\ & = & 2^{2l+D}{\left(\frac{(n-l-1)!}{2\pi (n+l+D-3)!}\right)}^{1/2}\Gamma \left(l+\frac{D-1}{2}\right)\;\eta^{\frac{D+1}{2}}, \end{array}$$

where η−L−1=n−l−1 and $L+1=l+\frac{D-1}{2}$. The symbols $C_{m}^{\left(\alpha \right)}\left(y\right)$ and ${\tilde{C}}_{m}^{\left(\alpha \right)}\left(y\right)$ denote the orthogonal and orthonormal Gegenbauer polynomials [28] with respect to the weight function $\omega_{\alpha }^{*}\left(y\right)={\left(1-y^{2}\right)}^{\alpha -\frac{1}{2}}$ on the interval [−1,+1], respectively, which are mutually related by:

(15) $${\tilde{C}}_{k}^{\left(\lambda \right)}\left(x\right)={\left(\frac{k!\left(k+\lambda \right)\Gamma^{2}\left(\lambda \right)}{\pi 2^{1-2\lambda }\Gamma \left(2\lambda +k\right)}\right)}^{1/2}C_{k}^{\left(\lambda \right)}\left(x\right),$$

so that

$${\tilde{C}}_{n-l-1}^{\left(l+\frac{D-1}{2}\right)}\left(x\right)=A{\left(n,l;D\right)}^{\frac{1}{2}}\;\;C_{n-l-1}^{\left(l+\frac{D-1}{2}\right)}\left(x\right)$$

with the constant

(16) $$A\left(n,l;D\right)=\frac{\left(n-l-1\right)!\left(n+\frac{D-3}{2}\right){\left[\Gamma \left(l+\frac{D-1}{2}\right)\right]}^{2}}{2^{2-2l-D}\pi \Gamma \left(n+l+D-2\right)}.$$

The position and momentum *D*-dimensional wavefunctions (3) and (12), respectively, reduce to the corresponding three-dimensional wavefunctions obtained by numerous authors (see e.g., [43,44,45]).

### Multidimensional Probability Densities

Then, the corresponding position and momentum probability densities of a *D*-dimensional hydrogenic system (i.e., the charge and momentum densities of the system) are given in terms of the hyperquantum numbers, (n,l,{μ}), by:

(17) $$\begin{array}{ccc} \rho_{n,l,\{\mu \}}\left(\vec{r}\right) & = & {\left|\Psi_{n,l,\{\mu \}}\left(\vec{r}\right)\right|}^{2}=R_{nl}^{2}\left(r\right)\times {\left|Y_{l,\{\mu \}}\left(\Omega_{D-1}\right)\right|}^{2}\\ & = & K_{n,l}^{2}{\tilde{r}}^{2l}e^{-\tilde{r}}{\left[L_{n-l-1}^{\left(2l+D-2\right)}\left(\tilde{r}\right)\right]}^{2}\times {\left|Y_{l,\{\mu \}}\left(\Omega_{D-1}\right)\right|}^{2}\\ & = & {\left(\frac{2Z}{\eta }\right)}^{D}\frac{1}{2\eta }\;\;\frac{\omega_{2L+1}\left(\tilde{r}\right)}{{\tilde{r}}^{D-2}}\;\;{\left[{\tilde{L}}_{\eta -L-1}^{\left(2L+1\right)}\left(\tilde{r}\right)\right]}^{2}\times {\left|Y_{l,\{\mu \}}\left(\Omega_{D-1}\right)\right|}^{2} \end{array}$$

(18) $$\begin{array}{ccc} & \equiv & \rho_{n,l}\left(\tilde{r}\right)\times {\left|Y_{l,\{\mu \}}\left(\Omega_{D-1}\right)\right|}^{2}, \end{array}$$

in position space, and

(19) $$\begin{array}{ccc} \gamma_{n,l,\{\mu \}}\left(\vec{p}\right) & = & {\left|{\tilde{\Psi }}_{n,l,\{\mu \}}\left(\vec{p}\right)\right|}^{2}=M_{n,l}^{2}\left(p\right)\times {\left|Y_{l,\{\mu \}}\left(\Omega_{D-1}\right)\right|}^{2}\\ & = & K_{n,l}^{\prime 2}\frac{{\left(\eta \tilde{p}\right)}^{2l}}{{\left(1+\eta^{2}{\tilde{p}}^{2}\right)}^{2L+4}}{\left[C_{\eta -L-1}^{\left(L+1\right)}\left(\frac{1-\eta^{2}{\tilde{p}}^{2}}{1+\eta^{2}{\tilde{p}}^{2}}\right)\right]}^{2}\times {\left|Y_{l,\{\mu \}}\left(\Omega_{D-1}\right)\right|}^{2}\\ & = & {\left(\frac{\eta }{Z}\right)}^{D}{\left(1+y\right)}^{3}{\left(\frac{1+y}{1-y}\right)}^{\frac{D-2}{2}}\omega_{L+1}^{*}\left(y\right)\;\;{\left[{\tilde{C}}_{\eta -L-1}^{\left(L+1\right)}\left(y\right)\right]}^{2}\times {\left|Y_{l,\{\mu \}}\left(\Omega_{D-1}\right)\right|}^{2} \end{array}$$

(20) $$\begin{array}{ccc} & \equiv & \gamma_{n,l}\left(\tilde{p}\right)\times {\left|Y_{l,\{\mu \}}\left(\Omega_{D-1}\right)\right|}^{2}, \end{array}$$

in momentum space. The symbols $\rho_{n,l}\left(\tilde{r}\right)$ and $\gamma_{n,l}\left(\tilde{p}\right)$ denote the radial densities:

(21) $$\begin{array}{ccc} \rho_{n,l}\left(\tilde{r}\right) & = & {\left[R_{n,l}\left(r\right)\right]}^{2}=\frac{\lambda^{-D}}{2\eta }\;\;\frac{\omega_{2L+1}\left(\tilde{r}\right)}{{\tilde{r}}^{D-2}}\;\;{\left[{\tilde{L}}_{\eta -L-1}^{2L+1}\left(\tilde{r}\right)\right]}^{2} \end{array}$$

and

(22) $$\begin{array}{ccc} \gamma_{n,l}\left(\tilde{p}\right) & = & {\left[M_{n,l}\left(p\right)\right]}^{2}={\left(\frac{\eta }{Z}\right)}^{D}{\left(1+y\right)}^{3}{\left(\frac{1+y}{1-y}\right)}^{\frac{D-2}{2}}\omega_{L+1}^{*}\left(y\right)\;\;{\left[{\tilde{C}}_{\eta -L-1}^{\left(L+1\right)}\left(y\right)\right]}^{2}, \end{array}$$

in position and momentum spaces, respectively.

## 3. Heisenberg-like Measures of High-Dimensional Hydrogenic States

In this section, we show and discuss the Heisenberg-like uncertainty measures of the high-dimensional (D→∞) hydrogenic states in position and momentum spaces. They are given by the radial position and momentum expectation values of the corresponding probability distributions for such states. First, we give the somewhat highbrow, but compact, corresponding expressions, for any *D*-dimensional hydrogenic state with D≥2 by means of a generalized hypergeometric function of ${}_{q}F_{p}\left(z\right)$ type, and then we obtain the high dimensional limit by asymptotical techniques of integral functionals of the orthogonal polynomials involved in the associated hydrogenic wavefunctions.

### 3.1. Position Space

The Heisenberg-like uncertainty measures of the *D*-dimensional hydrogenic states in position space are given by the expectation values:

(23) $$\begin{array}{ccc} \langle r^{\alpha }\rangle & = & \int_{R_{D}}r^{\alpha }\rho \left(\vec{r}\right)d\vec{r}=\int_{0}^{\infty }r^{\alpha +D-1}R_{n,l}^{2}\left(r\right)dr\\ & = & \frac{1}{2\eta }{\left(\frac{\eta }{2Z}\right)}^{\alpha }\int_{0}^{\infty }\omega_{2l+D-2}\left(t\right)\;\;{\left[{\tilde{L}}_{n-l-1}^{\left(2l+D-2\right)}\left(t\right)\right]}^{2}\;t^{\alpha +1}\;d\;t, \end{array}$$

which holds for α>−D−2l. Then, using the integral representation of the generalized hypergeometric functions ${}_{p+1}F_{p}\left(1\right)$ [28,46], one finds [38,47,48,49,50] that

(24) $$\begin{array}{c} \langle r^{\alpha }\rangle =\frac{\eta^{\alpha -1}}{2^{\alpha +1}Z^{\alpha }}\frac{\Gamma (2L+\alpha +3)}{\Gamma (2L+2)}\;{\;}_{3}F_{2}\left(\left.\begin{array}{cc} -\eta +L+1, & -\alpha -1,\;\alpha +2\\ & 2L+2,\;1 \end{array}\right|1\right), \end{array}$$

where the symbol ${}_{p+1}F_{p}\left(z\right)$ denotes the generalized hypergemetric series given by

(25) $${}_{p+1}F_{p}\left(\left.\begin{array}{c} a_{1},\ldots ,a_{p+1}\\ b_{1},\ldots ,b_{p} \end{array}\right|z\right)=\sum_{j=0}^{\infty }\frac{{\left(a_{1}\right)}_{j}\cdots {\left(a_{p+1}\right)}_{j}}{{\left(b_{1}\right)}_{j}\cdots {\left(b_{p}\right)}_{j}}\frac{z^{j}}{j!},$$

which is terminating when the first one or more of the top parameters is a nonnegative integer, so that it reduces to a polynomial in *z*. Then, note that the previous functions ${}_{3}F_{2}\left(1\right)$ given by Equation (24) are terminating hypergeometric functions (so, a polynomial) evaluated at z=1. An alternative expression could be obtained via the not yet explored method of Perrey et al. [51]. From (23) or (24) and the known properties of the function ${}_{3}F_{2}\left(1\right)$ [28] one obtains $\langle r^{0}\rangle =1$ and the first few Heisenberg-like measures. Moreover, for the ground state (n=1,l=0) of the *D*-dimensional hydrogenic state one has

(26) $$\begin{array}{c} \langle r^{\alpha }\rangle ={\left(\frac{D-1}{4Z}\right)}^{\alpha }\frac{\Gamma (D+\alpha )}{\Gamma (D)};\;\alpha >-D. \end{array}$$

For further information and reduction to three-dimensional values, see [25].

The Heisenberg-like uncertainty measures of the high-dimensional hydrogenic states can be obtained by means of the D→+∞ limit of Equation (24). Then, taking into account the asymptotics:

(27) $${}_{3}F_{2}\left(-n+l+1,-\alpha -1,\alpha +2;2l-1+D,1;1\right)=1+\frac{\left(\alpha +1)(\alpha +2)(n-l-1\right)}{2l-1+D}+O\left(\frac{1}{D^{2}}\right).$$

Together with the following asymptotics of the gamma ratio (see e.g., Equations (5), (11) and (12) in [28]),

(28) $$\frac{\Gamma (D+2l+\alpha )}{\Gamma (D+2l-1)}=D^{1+\alpha }\left(1+\frac{\left(\alpha +1)(\alpha +4l-2\right)}{2D}+O\left(\frac{1}{D^{2}}\right)\right),$$

we finally have [26] the values:

(29) $$\begin{array}{ccc} \langle r^{\alpha }\rangle & = & {\left(\frac{D^{2}}{4Z}\right)}^{\alpha }\left(1+\frac{\left(\alpha +1)(\alpha +4l-2\right)}{2D}\right)\left(1+\frac{\left(\alpha +1)(\alpha +2)(n-l-1\right)}{D}\right)\left(1+O\left(\frac{1}{D^{2}}\right)\right), \end{array}$$

for the Heisenberg-like uncertainty measures of the high-dimensional hydrogenic states. Note that in this limit one has that $\langle r^{\alpha }\rangle \to r_{char}^{\alpha }$, where $r_{char}=\frac{D^{2}}{4Z}$. Then, $r_{char}$ appears to be as the characteristic length for the Coulomb problem.

In particular, for l=n−1 the last expression yields the following values:

(30) $${\langle r^{\alpha }\rangle }_{cs}={\left(\frac{D^{2}}{4Z}\right)}^{\alpha }\left[1+\frac{\left(\alpha +1)(4n+\alpha -6\right)}{2D}\right]\left(1+O\left(\frac{1}{D^{2}}\right)\right),$$

for the position Heisenberg-like measures of the high dimensional hydrogenic states of circular type. Moreover, remark from this expression that the position Heisenberg-like expectation values for the ground state (n=1) of the *D*-dimensional hydrogenic system at high *D* are ${\langle r^{\alpha }\rangle }_{gs}={\left(\frac{D^{2}}{4Z}\right)}^{\alpha }$, in agreement with the corresponding limit of (26).

### 3.2. Momentum Space

The Heisenberg-like uncertainty measures of the *D*-dimensional hydrogenic states (n,l,{μ}) in momentum space are given by the expectation values,

(31) $$\begin{array}{ccc} \langle p^{\alpha }\rangle & = & \int p^{\alpha }\gamma \left(\vec{p}\right)d\vec{p}=\int_{0}^{\infty }p^{\alpha +D-1}M_{n,l}^{2}\left(p\right)dp\\ & = & {\left(\frac{Z}{\eta }\right)}^{\alpha }K_{n,l}\int_{-1}^{+1}{\left[C_{k}^{\left(\nu \right)}\left(t\right)\right]}^{2}{\left(1-t\right)}^{\nu +\frac{\alpha -1}{2}}{\left(1+t\right)}^{\nu -\frac{\alpha -1}{2}}dt \end{array}$$

(32) $$\begin{array}{ccc} & = & {\left(\frac{Z}{\eta }\right)}^{\alpha }\int_{-1}^{+1}\omega_{\nu }^{*}\left(t\right){\left[{\tilde{C}}_{k}^{\left(\nu \right)}\left(t\right)\right]}^{2}{\left(1-t\right)}^{\frac{\alpha }{2}}{\left(1+t\right)}^{1-\frac{\alpha }{2}}dt, \end{array}$$

(which holds for −2l−D≤α≤2l+D+2). Note that k=η−L−1=n−l−1, ν=L+1=l+(D−1)/2, $\omega_{\nu }^{*}\left(t\right)={\left(1-t^{2}\right)}^{\nu -\frac{1}{2}}={\left(1-t^{2}\right)}^{l+\frac{D-2}{2}}$ is the weight function of the Gegenbauer polynomials ${\tilde{C}}_{k}^{\left(\nu \right)}\left(t\right)$, and the constant:

(33) $$\begin{array}{ccc} K_{n,l} & = & \frac{K_{n,l}^{\prime 2}}{2^{2l+D+1}\eta^{D}}=\;2^{2(L+1)}\eta {\left[\Gamma (L+1)\right]}^{2}\left(\frac{(\eta -L-1)!}{2\pi (\eta +L)!}\right). \end{array}$$

Then, the use of the properties of the function ${}_{5}F_{4}\left(z\right)$ allows us to find

(34) $$\begin{array}{c} \langle p^{\alpha }\rangle =\frac{2^{1-2\nu }Z^{\alpha }\sqrt{\pi }}{k!\eta^{\alpha }}\frac{\left(k+\nu \right)\Gamma \left(k+2\nu \right)\Gamma \left(\nu +\frac{\alpha +1}{2}\right)\Gamma \left(\nu +\frac{3-\alpha }{2}\right)}{\Gamma^{2}\left(\nu +\frac{1}{2}\right)\Gamma \left(\nu +1\right)\Gamma \left(\nu +\frac{3}{z}\right)}\\ \times {\;}_{5}F_{4}\left(\left.\begin{array}{cc} -k, & k+2\nu ,\nu ,\nu +\frac{\alpha +1}{2},\nu +\frac{3-\alpha }{2}\\ & 2\nu ,\nu +\frac{1}{2},\nu +1,\nu +\frac{3}{2} \end{array}\right|1\right), \end{array}$$

for the momentum Heisenberg-like measures [52] of a generic *D*-dimensional hydrogenic state (n,l). From (32) or (34) and the known properties of the function ${}_{5}F_{4}\left(1\right)$ [28] one obtains $\langle p^{0}\rangle =1$ and the first few Heisenberg-like measures.

For further information and reduction to three-dimensional values, see [25]. In particular, let us remark that the problem to calculate explicitly the momentum Heisenberg-like measures with an odd integer order for the *D*-dimensional hydrogenic system requires a more delicate treatment as is detailed in Section 4 of [25]. Note also that for the ground state (n=1,l=0) of the *D*-dimensional hydrogenic state one has the value:

(35) $$\begin{array}{c} \langle p^{\alpha }\rangle ={\left(\frac{2Z}{D-1}\right)}^{\alpha }\frac{2\;\Gamma \left(\frac{D-\alpha }{2}+1\right)\Gamma \left(\frac{D+\alpha }{2}\right)}{D\;\Gamma^{2}\left(\frac{D}{2}\right)};\;-D<\alpha <D+2 \end{array}$$

for the momentum expectation values when D≥2.

The momentum Heisenberg-like uncertainty measures of the high-dimensional hydrogenic states can be obtained as follows by means of the D→+∞ limit of Equation (31) or (34). Using the definition (25) for the terminating hypergeometric function ${}_{5}F_{4}\left(z\right)$, one can [53] express (34) as:

(36) 〈pα〉ηαZα=2k!(k+ν)Γ(k+2ν)Γ(2ν+1)Γ(ν+α+12)Γ(ν+3−α2)Γ(ν+12)Γ(ν+32)×∑j=0k(−1)jkj(k+2ν)j(ν)j(ν+α+12)j(ν+3−α2)j(2ν)j(ν+1)j(ν+12)j(ν+32)j.

In the ν→+∞ limit we find the asymptotical formula:

(37) $$\langle p^{\alpha }\rangle =\frac{Z^{\alpha }}{\eta^{\alpha }}\left(1+\frac{\alpha (\alpha -2)(2k+1)}{4\nu }+o\left(1/\nu \right)\right),\;\nu \to +\infty .$$

And taking into account that $\eta =n+\frac{D-3}{2}$ and $\nu =l+\frac{D-1}{2}$, one finally obtains [25] the values:

(38) $$\begin{array}{ccc} \langle p^{\alpha }\rangle & = & {\left(\frac{Z}{n+\frac{D-3}{2}}\right)}^{\alpha }\left(1+\frac{\alpha (\alpha -2)(2n-2l-1)}{2D}+O\left(D^{-2}\right)\right)\\ & = & {\left(\frac{2Z}{D}\right)}^{\alpha }\left(1+\frac{\alpha (\alpha -2)(2n-2l-1)}{2D}+O\left(D^{-2}\right)\right) \end{array}$$

for the high dimensional (D→∞) hydrogenic states (n,l,{μ}), which is valid at −2l−D≤α≤2l+D+2. Note that in such a limit, one has that ${\left(\frac{D^{2}}{4Z}\right)}^{-\alpha }\langle p^{\alpha }\rangle \to 1$. Thus, our *D*-dimensional hydrogenic system has a characteristic momentum, $p_{char}=\frac{2Z}{D}$, which corresponds to the localization of the maximum of the ground-state probability density in momentum space. So, the high-dimensional hydrogenic system seems to behave like an electron moving with velocity $p_{char}$ in a circular orbit of angular momentum $\frac{D}{2}$ and radius $r_{char}$. See [25] for Heisenberg-like measures with a specific α.

In particular, for l=n−1 the last expression yields the following values:

(39) $${\langle p^{\alpha }\rangle }_{cs}={\left(\frac{2Z}{D}\right)}^{\alpha }\left(1+\frac{\alpha (\alpha -2)}{2D}+O\left(D^{-2}\right)\right),$$

for the momentum Heisenberg-like measures of the high dimensional hydrogenic states of circular type. Moreover, remark from this expression that the momentum Heisenberg-like expectation values for the ground state (n=1) of the high-dimensional hydrogenic state are ${\langle p^{\alpha }\rangle }_{gs}={\left(\frac{2Z}{D}\right)}^{\alpha }$, in agreement with the corresponding limit of (35).

### 3.3. Heisenberg-like Uncertainty Relations

Then, from Equations (29) and (38) one has that the position–momentum Heisenberg product $\langle r^{\alpha }\rangle \langle p^{\alpha }\rangle$ for the high dimensional (D→∞) hydrogenic states (n,l,{μ}) fulfills:

(40) $$\begin{array}{c} \langle r^{\alpha }\rangle \langle p^{\alpha }\rangle ∼{\left(\frac{D}{2}\right)}^{\alpha }\left(1+\frac{\left(\alpha +1)(\alpha +4l-2\right)}{2D}\right)\left(1+\frac{\left(\alpha +1)(\alpha +2)(n-l-1\right)}{D}\right)\\ \times \left(1+\frac{\alpha (\alpha -2)(2n-2l-1)}{2D}\right), \end{array}$$

valid for α∈(−D−2l,D+2l+2); or, more simply [53]

(41) $$\langle r^{\alpha }\rangle \langle p^{\alpha }\rangle ={\left(\frac{D}{2}\right)}^{\alpha }\left(1+O\left(\frac{1}{D^{}}\right)\right).$$

For the high dimensional hydrogenic states of circular type (i.e., when l=n−1) we have:

(42) $$\begin{array}{ccc} {\langle r^{\alpha }\rangle }_{cs}{\langle p^{\alpha }\rangle }_{cs} & ∼ & {\left(\frac{D}{2}\right)}^{\alpha }\left(1+\frac{\left(\alpha +1)(\alpha +4n-6)+\alpha (\alpha -2\right)}{2D}+\frac{\alpha (\alpha -2)(\alpha +1)(\alpha +4n-6)}{4D^{2}}\right), \end{array}$$

which, for α=2 boils down to:

(43) $${\langle r^{2}\rangle }_{cs}{\langle p^{2}\rangle }_{cs}=\frac{D^{2}}{4}\left[1+\frac{6(n-1)}{D}\right]\left(1+o(1)\right).$$

Then, we have that ${\langle r^{2}\rangle }_{gs}{\langle p^{2}\rangle }_{gs}=\frac{D^{2}}{4}$ for the ground state (n=1). Then, in high dimensions, the standard Heisenberg uncertainty relation (first found by Heisenberg and Kennard for one-dimensional systems [54,55]):

(44) $${\langle r^{2}\rangle }_{gs}{\langle p^{2}\rangle }_{gs}\geq \frac{D^{2}}{4}$$

saturates not only for the oscillator ground state but also at the hydrogenic ground state. Moreover, the expression (40) for α=2 also fulfills the uncertainty relation:

(45) $$\langle r^{2}\rangle \langle p^{2}\rangle \geq {\left(l+\frac{D}{2}\right)}^{2},$$

which holds for general spherically-symmetric potentials [56]. Moreover, the high-dimensional uncertainty relations also satisfy the corresponding Heisenberg-like relation for general quantum systems of Angulo [57,58] (see also [59]). For further details and recent information about the position and momentum of Heisenberg-like uncertainty measures of multidimensional hydrogenic systems and their associated uncertainty relations, see [25,53,59,60].

Interestingly, the high dimensional hydrogenic behavior (41) has also been observed for the high dimensional Heisnberg-like product of the the high dimensional harmonic (oscillator-like) systems [60,61]. Since the Coulomb and quadratic potentials of the hydrogenic and harmonic systems, respectively, are so different we can possibly conjecture that the expression (41) remains valid for high dimensional states of quantum systems with a general power-law central potential.

## 4. Rényi and Shannon Entropies of High-Dimensional Hydrogenics States

In this section, we show and revise the Rényi and Shannon entropies of the high-dimensional (D→∞) hydrogenic states (n,l,{μ}) in position and momentum spaces. Recently, the position and momentum Rényi entropies [27,62,63] and the position Shannon entropies [26,64] for *D*-dimensional hydrogenic states (n,l,{μ}) with D≥2. However, the resulting general expressions are given in a closed but highbrow and not easily handy way, so that they can provide the values of these physical entropies only algorithmically. Here we obtain the high dimensional limit in a closed, compact analytical form by use of asymptotical techniques of integral functionals of the orthogonal polynomials involved in the associated hydrogenic wavefunctions [33].

### 4.1. Position Space

In position space, the Rényi entropies $R_{q}\left[\rho \right],q>0$ of a *D*-dimensional hydrogenic state (n,l,{μ}) is defined [27,62] as:

(46) $$R_{q}\left[\rho_{n,l,\{\mu \}}\right]=\frac{1}{1-q}\log\int_{R^{D}}{\left[\rho_{n,l,\{\mu \}}\left(\vec{r}\right)\right]}^{q}\;d\vec{r},\;q\neq 1,$$

where the state’s probability density $\rho_{n,l,\{\mu \}}$ is given by (17), and the volume element is:

$$d\vec{r}=r^{D-1}dr\;d\Omega_{D-1},\;d\Omega_{D-1}=\left(\prod_{j=1}^{D-2}\sin^{2\alpha_{j}}\theta_{j}\right)d\phi ,$$

with $2\alpha_{j}=D-j-1$. Then, this expression can be decomposed as:

(47) $$\begin{array}{ccc} R_{q}\left[\rho_{n,l,\{\mu \}}\right] & = & R_{q}\left[\rho_{n,l}\right]+R_{q}\left[Y_{l,\{\mu \}}\right], \end{array}$$

where the symbols $R_{p}\left[\rho_{n,l}\right]$ and $R_{p}\left[Y_{l,\{\mu \}}\right]$ defined by

(48) $$R_{q}\left[\rho_{n,l}\right]=\frac{1}{1-q}\log\int_{0}^{\infty }{\left[\rho_{n,l}\left(r\right)\right]}^{q}r^{D-1}\;dr,$$

(49) $$R_{q}\left[Y_{l,\{\mu \}}\right]=\frac{1}{1-q}\log\Lambda_{l,\{\mu \}}\left(\Omega_{D-1}\right),$$

denote the radial and angular parts of the Rényi entropy $R_{q}\left[\rho_{n,l,\{\mu \}}\right]$, respectively, and with the following Rényi-like integral functional of the hyperspherical harmonics:

(50) $$\begin{array}{ccc} \Lambda_{l,\{\mu \}}\left(\Omega_{D-1}\right) & = & \int_{S^{D-1}}{\left|Y_{l,\{\mu \}}\left(\Omega_{D-1}\right)\right|}^{2q}\;d\Omega_{D-1}. \end{array}$$

Now we calculate the high dimensional limit of these quantities. To tackle the high dimensional limit of the radial Rényi entropy $R_{q}\left[\rho_{n,l}\right]$, we first consider the $L_{q}$-norm of the Laguerre polynomials:

(51) $$N_{n}\left(\alpha ,q,\beta \right)=\int_{0}^{\infty }{\left({\left[{\tilde{L}}_{n}^{\left(\alpha \right)}\left(x\right)\right]}^{2}\;w_{\alpha }\left(x\right)\right)}^{q}\;x^{\beta }\;dx\;,\;\alpha >-1\;\;,q>0\;\;,\beta +q\alpha >-1,$$

so that, from Equation (48), one can express the radial Rényi entropy in the form:

(52) $$R_{q}\left[\rho_{n,l}\right]=\frac{1}{1-q}\log\;\left[\frac{\eta^{D(1-q)-q}}{2^{D(1-q)+q}Z^{D(1-q)}}N_{n,l}\left(D,q\right)\right],$$

where $N_{n,l}\left(D,p\right)=N_{n}\left(\alpha ,p,\beta \right)$, with $\tilde{r}\equiv x$ and

(53) α=2L+1=2l+D−2,l=0,1,2,…,n−1,q>0andβ=(2−D)q+D−1,

which guarantee the convergence of integral (51); that is, the condition β+qα=2lq+D−1>−1 is always satisfied for physically meaningful values of the parameters. Moreover, the norm $N_{n,l}\left(D,q\right)$ can be rewritten as:

(54) $$\begin{array}{ccc} N_{n,l}\left(D,q\right) & = & \int_{0}^{\infty }{\left({\left[{\tilde{L}}_{n-l-1}^{\left(D+2l-2\right)}\left(x\right)\right]}^{2}\;w_{D+2l-2}\left(x\right)\right)}^{q}\;x^{2q-1+(1-q)D}\;dx\\ & = & {\left[\frac{\Gamma (n-l)}{\Gamma (n+l+D-2)}\right]}^{q}\int_{0}^{\infty }x^{D+2lq-1}e^{-qx}{\left[L_{n-l-1}^{\left(D+2l-2\right)}\left(x\right)\right]}^{2q}\;dx. \end{array}$$

According to Equations (52) and (54), the determination of the high dimensional limit of the radial Rényi entropy boils down to finding the corresponding limit of the integral functional involved in the norm $N_{n,l}\left(D,q\right)$. The latter can be obtained by use of Theorem 1 of Temme et al. [33,34], so that for every non-negative q≠1 one has

(55) $$\begin{array}{ccc} N_{n,l}\left(D,q\right) & ∼ & \frac{{\left(2\pi \right)}^{\frac{1-q}{2}}{\left|q-1\right|}^{2(n-l-1)q}}{\Gamma {\left(n-l\right)}^{q}}q^{-2q(n-1)}{\left(\frac{D}{e}\right)}^{D(1-q)}q^{-D}D^{q\left(n-l+\frac{1}{2}\right)-\frac{1}{2}}, \end{array}$$

where we have also used the Stirling’s formula [28] for the gamma function $\Gamma \left(x\right)=e^{-x}x^{x-\frac{1}{2}}{\left(2\pi \right)}^{\frac{1}{2}}\left[1+O\left(x^{-1}\right)\right]$. Thus, from (52) and (55) we find [33] the following high-*D* behavior for the radial Rényi entropy:

(56) $$R_{q}\left[\rho_{n,l}\right]∼2D\log\left[D\right]+D\log\left[\frac{q^{\frac{1}{q-1}}}{4Ze}\right]+\frac{q\left(n-l-\frac{1}{2}\right)-\frac{1}{2}}{1-q}\log D+\frac{1}{1-q}\log F\left(n,l,q\right),$$

where $F\left(n,l,q\right)=\frac{{\left(2\pi \right)}^{\frac{1-q}{2}}{\left|q-1\right|}^{2(n-l-1)q}}{\Gamma {\left(n-l\right)}^{q}}\frac{e^{\left(2n-3)(1-q\right)}}{q^{2q(n-1)}}$.

Let us now tackle the high dimensional limit of the angular Rényi entropy $R_{q}\left[Y_{l,\{\mu \}}\right]$ given by Equations (4), (50) and (49). Then, using Theorem 2 at zeroth-order of Temme et al. [33,34] relative to some related Rényi-like integral functionals of Gegenbauer polynomials, we obtain the expression:

(57) $$\begin{array}{ccc} R_{q}\left[Y_{l,\{\mu \}}\right] & ∼ & \frac{1}{1-q}\log\left(\frac{\Gamma {\left(\frac{D}{2}+l\right)}^{q}}{\Gamma \left(\frac{D}{2}+ql\right)}\right)+\frac{D}{2}\log\pi \\ & & +\frac{1}{1-q}\log\left(\tilde{E}{\left(D,\left\{\mu \right\}\right)}^{q}\tilde{M}\left(D,q,\left\{\mu \right\}\right)\frac{\Gamma (1+q\mu_{D-1})}{\Gamma {\left(1+\mu_{D-1}\right)}^{q}}2^{1-q}\right)\\ & ∼ & -\log\left(\Gamma \left(\frac{D}{2}\right)\right)+\frac{D}{2}\log\pi +\frac{1}{1-q}\log\left(\tilde{E}{\left(D,\left\{\mu \right\}\right)}^{q}\tilde{M}\left(D,q,\left\{\mu \right\}\right)\frac{\Gamma (1+q\mu_{D-1})}{\Gamma {\left(1+\mu_{D-1}\right)}^{q}}2^{1-q}\right)\\ & ∼ & -\frac{D}{2}\log D+\frac{D}{2}\log\left(2\pi e\right)+\frac{1}{2}\log D\\ & & +\frac{1}{1-q}\log\left(\tilde{E}{\left(D,\left\{\mu \right\}\right)}^{q}\tilde{M}\left(D,q,\left\{\mu \right\}\right)\frac{\Gamma (1+q\mu_{D-1})}{\Gamma {\left(1+\mu_{D-1}\right)}^{q}}{\left(\frac{\pi }{2}\right)}^{\frac{q-1}{2}}\right), \end{array}$$

where

(58) $$\tilde{M}\left(D,q,\left\{\mu \right\}\right)\equiv 4^{q(l-\mu_{D-1})}\pi^{1-\frac{D}{2}}\prod_{j=1}^{D-2}\frac{\Gamma \left(q\left(\mu_{j}-\mu_{j+1}\right)+\frac{1}{2}\right)}{\Gamma {\left(\mu_{j}-\mu_{j+1}+1\right)}^{q}}$$

and

(59) $$\tilde{E}\left(D,\left\{\mu \right\}\right)\equiv \prod_{j=1}^{D-2}\frac{{\left(\alpha_{j}+\mu_{j+1}\right)}^{2(\mu_{j}-\mu_{j+1})}}{{\left(2\alpha_{j}+2\mu_{j+1}\right)}_{\mu_{j}-\mu_{j+1}}}\frac{1}{{\left(\alpha_{j}+\mu_{j+1}\right)}_{\mu_{j}-\mu_{j+1}}}.$$

From Equations (56)–(59) and (47) we find [33] that the total Rényi entropy $R_{q}\left[\rho_{n,l,\{\mu \}}\right]$ in position space for a high-dimensional hydrogenic-state with the hyerquantum numbers (n,l,{μ}), has the expression:

(60) $$\begin{array}{ccc} R_{q}\left[\rho_{n,l,\{\mu \}}\right] & ∼ & \log\left(\frac{D^{2D}}{\Gamma \left(\frac{D}{2}\right)}\right)+D\log\left(\frac{q^{\frac{1}{q-1}}\sqrt{\pi }}{4Ze}\right)+\frac{q\left(n-l-\frac{1}{2}\right)-\frac{1}{2}}{1-q}\log D\\ & & +\frac{1}{1-q}\log\left(\tilde{E}{\left(D,\left\{\mu \right\}\right)}^{q}\tilde{M}\left(D,q,\left\{\mu \right\}\right)F\left(n,l,q\right)\;\frac{\Gamma (1+q\mu_{D-1})}{\Gamma {\left(1+\mu_{D-1}\right)}^{q}}\;{\left(\frac{\pi }{2}\right)}^{\frac{q-1}{2}}\right)\\ & ∼ & \frac{3}{2}D\log D+D\log\left(\frac{q^{\frac{1}{q-1}}}{Z}\sqrt{\frac{\pi }{8e}}\right)+\frac{q(n-l-1)}{1-q}\log D\\ & & +\frac{1}{1-q}\log\left(\tilde{E}{\left(D,\left\{\mu \right\}\right)}^{q}\tilde{M}\left(D,q,\left\{\mu \right\}\right)F\left(n,l,q\right)\;\frac{\Gamma (1+q\mu_{D-1})}{\Gamma {\left(1+\mu_{D-1}\right)}^{q}}\;{\left(\frac{\pi }{2}\right)}^{\frac{q-1}{2}}\right), \end{array}$$

which holds for every non-negative q≠1. Then, after some algebraic and asymptotical manipulations, we finally have the value:

(61) $$\begin{array}{ccc} R_{q}\left[\rho_{n,l,\{\mu \}}^{\left(H\right)}\right] & = & \frac{3}{2}D\log\left(\frac{D}{2}\right)+D\log\left(\frac{q^{\frac{1}{q-1}}}{Z}\sqrt{\frac{\pi }{e}}\right)+\frac{q(n-l-1)}{1-q}\log D+O\left(1\right), \end{array}$$

for the Rényi entropies of the high-dimensional (D→∞) hydrogenic states (n,l,{μ}) in position space. See [33] for further details and application to various relevant classes of hydrogenic states, such as the *ns* and circular states. See [33] for further details and application to various relevant classes of hydrogenic states, such as the *ns* and circular states.

Moreover, from this expression we can calculate the Shannon entropy for the high dimensional stationary hydrogenic states with fixed hyperquantum numbers $\left(n,l,\left\{\mu \right\}\right)$ since this entropic measure is defined [63,64]) as:

(62) $$S\left[\rho_{n,l,\{\mu \}}\right]=-\int \rho_{n,l,\{\mu \}}\left(\vec{r}\right)\log\rho_{n,l,\{\mu \}}\left(\vec{r}\right)d\vec{r}=\lim_{q\to 1}R_{q}\left[\rho_{n,l,\{\mu \}}\right].$$

Since $q^{\frac{1}{q-1}}\to e$ when q→1, the expression (61) in this limit simplifies as:

(63) $$\begin{array}{ccc} S[\rho_{n,l,\{\mu \}}] & ∼ & \log\left(\frac{D^{2D}}{\Gamma \left(\frac{D}{2}\right)}\right)+D\log\left(\frac{\sqrt{\pi }}{4Z}\right)\\ & ∼ & \frac{3}{2}D\log D+D\log\left(\frac{\sqrt{e\pi }}{\sqrt{8}Z}\right),\;D\to \infty \end{array}$$

for the Shannon entropy of the high-dimensional hydrogenic states (n,l,{μ}) in position space. See [32] for further details and applications.

### 4.2. Momentum Space

In momentum space the Rényi entropies $R_{q}\left[\gamma_{n,l,\{\mu \}}\right],q>0$ of a *D*-dimensional hydrogenic state (n,l,{μ}) is defined [62] as:

(64) $$R_{q}\left[\gamma_{n,l,\{\mu \}}\right]=\frac{1}{1-q}\log\int_{R^{D}}{\left[\gamma_{n,l,\{\mu \}}\left(\vec{p}\right)\right]}^{q}\;d\vec{p},\;q\neq 1,$$

where $\gamma_{n,l,\{\mu \}}$ denotes the state’s probability density given by Equation (22), so that it can be expressed as:

(65) $$\begin{array}{ccc} R_{q}\left[\gamma_{n,l,\{\mu \}}\right] & = & R_{q}\left[\gamma_{n,l}\right]+R_{q}\left[Y_{l,\{\mu \}}\right], \end{array}$$

where the momentum radial Shannon entropy $R_{q}\left[\gamma_{n,l}\right]$ is defined by:

(66) $$R_{q}\left[\gamma_{n,l}\right]=\frac{1}{1-q}\log\int_{0}^{\infty }{\left[M_{n,l}\left(p\right)\right]}^{2q}p^{D-1}\;dp,$$

and the symbol $R_{q}\left[Y_{l,\{\mu \}}\right]$ denotes the abovementioned angular part of the Rényi entropy $R_{q}\left[\gamma_{n,l,\{\mu \}}\right]$ given by (49).

Working similarly as for position space, here we find the following expression:

(67) $$\begin{array}{ccc} R_{q}\left[\gamma_{n,l,\{\mu \}}\right] & ∼ & -\log\left(\frac{\eta^{D}\;\Gamma \left(\frac{D}{2}\right)}{Z^{D}}\right)+D\log\left(\sqrt{\pi }{\left(\frac{{\left(2q-1\right)}^{2q-1}}{q^{2q}}\right)}^{\frac{1}{2-2q}}\right)\\ & & +\frac{q\left(n-l-\frac{1}{2}\right)-\frac{1}{2}}{1-q}\log D+\frac{1}{1-q}\log(\tilde{E}{\left(D,\left\{\mu \right\}\right)}^{q}\tilde{M}\left(D,q,\left\{\mu \right\}\right){\bar{Q}}_{0}\left(q,n,l\right)\pi^{\frac{q-1}{2}})\\ & ∼ & -\frac{3}{2}D\log D+D\log\left(Z\sqrt{8e\pi }{\left(\frac{{\left(2q-1\right)}^{2q-1}}{q^{2q}}\right)}^{\frac{1}{2-2q}}\right)\\ & & +\frac{q(n-l-1)}{1-q}\log D+\frac{1}{1-q}\log(\tilde{E}{\left(D,\left\{\mu \right\}\right)}^{q}\tilde{M}\left(D,q,\left\{\mu \right\}\right){\bar{Q}}_{0}\left(q,n,l\right)\pi^{\frac{q-1}{2}}), \end{array}$$

(with q≠1 and $\eta ∼\frac{D}{2}$) for the high-*D* behavior of the total momentum Rényi entropy of the generic hydrogenic state (n,l,{μ}), where the symbols $\tilde{M}\left(D,q,\left\{\mu \right\}\right)$ and $\tilde{E}\left(D,\left\{\mu \right\}\right)$ are defined in Equations (58) and (59), respectively. Moreover, the factor $Q_{0}\left(q\right)$ is given by:

(68) $$Q_{0}\left(q,n,l\right)=\frac{\sqrt{2\pi }\;4^{q(n-l-1)}}{\Gamma {\left(n-l\right)}^{2q}}\frac{{\left(2q-1\right)}^{q\left(l+1\right)-\frac{1}{2}}{\left(q-1\right)}^{2q(n-l-1)}}{q^{q\left(2n-1\right)-\frac{1}{2}}}.$$

And with some algebraic and asymptotical manipulations, we have that:

(69) $$\begin{array}{ccc} R_{q}\left[\gamma_{n,l,\{\mu \}}^{\left(H\right)}\right] & = & -\frac{3}{2}D\log\left(\frac{D}{2}\right)+D\log\left(\frac{Z\sqrt{e\pi }}{{\tilde{q}}^{\frac{1}{q-1}}}\right)+\frac{q(n-l-1)}{1-q}\log D+O\left(1\right), \end{array}$$

where $\tilde{q}={\left(\frac{{\left(2q-1\right)}^{2q-1}}{q^{2q}}\right)}^{\frac{1}{2}}$. See [33] for further details and application to various relevant classes of hydrogenic states, such as the *ns* and circular states. Note from Equations (61) and (69) that the potential manifestation (i.e., the dependence on the nuclear charge *Z*) appears in the second term and the dependence on the quantum numbers (n,l) does not arise until the third term in both position and momentum Rényi entropies, respectively.

Moreover, from expression (69) we can calculate the momentum Shannon entropy for the high dimensional stationary hydrogenic states with fixed hyperquantum numbers $\left(n,l,\left\{\mu \right\}\right)$ since it is defined [63,64]) as

(70) $$S\left[\gamma_{n,l,\{\mu \}}\right]=-\int \gamma_{n,l,\{\mu \}}\left(\vec{r}\right)\log\gamma_{n,l,\{\mu \}}\left(\vec{r}\right)d\vec{r}=\lim_{q\to 1}R_{q}\left[\gamma_{n,l,\{\mu \}}\right].$$

Then, the expression (69) in the limit q→1 gives rise to the value:

(71) $$\begin{array}{c} S\left[\gamma_{n,l,\{\mu \}}\right]∼-\frac{3}{2}D\log D+D\log\left(Z\sqrt{8e\pi }\right), \end{array}$$

for the Shannon entropy of the high-dimensional (D→∞) hydrogenic states (n,l,{μ}) in momentum space.

### 4.3. Entropic Uncertainty Relations

Finally, from Equations (60) and (67), it is straightforward to obtain the leading term for the position–momentum Rényi-entropy-based uncertainty sum of the high-dimensional hydrogenic states for a pair of parameters *p* and *q* which fulfill the Holder conjugacy relation $\frac{1}{p}+\frac{1}{q}=2$. Indeed, the summation of the previously-found position and momentum Rényi entropies yields:

(72) $$\begin{array}{ccc} R_{q}\left[\rho_{n,l,\{\mu \}}\right]+R_{p}\left[\gamma_{n,l,\{\mu \}}\right] & ∼ & D\log\left[\pi {\left(\frac{{\left(2p-1\right)}^{\left(2p-1\right)}}{p^{2p}}\right)}^{\frac{1}{2-2p}}q^{\frac{1}{q-1}}\right]\\ & = & D\log\left[2\pi \;{\left(2p\right)}^{\frac{1}{2p-2}}\;{\left(2q\right)}^{\frac{1}{2q-2}}\right],\;q\neq 1,\;D\to \infty , \end{array}$$

for all states with fixed hyperquantum numbers (n,l,{μ}). Note that this expression saturates the known position-momentum Rényi-entropy-based uncertainty relation [65,66]. It is also interesting to point out that the dependence at second order on the quantum numbers *n* and *l* of this sum, numerically observed out of the so called conjugacy curve (i.e., for arbitrary positive pairs of values of *p* and *q*) can be explained by means of the second-order terms of the position and momentum Rényi entropies mentioned above.

Additionally, taking into account the expressions (63) and (71) for the Shannon entropy in the two conjugated position and momentum spaces, respectively, we have the following joint position-momentum Shannon entropy of the high dimensional hydrogenic states:

(73) $$\begin{array}{ccc} S\left[\rho_{n,l,\{\mu \}}\right]+S\left[\gamma_{n,l,\{\mu \}}\right] & ∼ & D\log\left[2\pi e\right], \end{array}$$

which saturates the position–momentum Shannon-entropy-based uncertainty relation [67], valid for general quantum systems.

Interestingly, the high dimensional hydrogenic behaviors (72) and (73) have also been observed for the high dimensional Rényi and Shannon entropic sums, respectively, of the harmonic (oscillator-like) systems [33,60]. Thus, here again we can possibly conjecture that the expressions (72) and (73) remain valid for high dimensional states of quantum systems with a general spherically-symmetric potential.

## 5. Conclusions

In conclusion, we have revised and reviewed the recent knowledge on the Heisenberg-like and entropy-like uncertainty measures of the high-dimensional (or pseudoclassical) atomic states of hydrogenic type, and their associated uncertainty relations. These quantities are shown to have a simple and compact analytical expression in terms of the space dimensionality *D*, the Coulomb strength or nuclear charge *Z*, and the principal and orbital hyperquantum numbers (n,l). The leading term of the position and momentum Heisenberg-like measures depends on (D,Z) only, so that the Heisenberg-like uncertainty product $\langle r^{\alpha }\rangle \langle p^{\alpha }\rangle ∼{\left(\frac{D}{2}\right)}^{\alpha }$ at first order. The leading term of the position and momentum entropic measures only depends on *D*, so that the entropy-like of Rényi and Shannon types behave as $\pm \frac{3}{2}D\log D$, respectively. Moreover, the dependence on (Z,n,l) arises at the second and third asymptotical orders in *D*, respectively. All this happens in such a way that the joint position-momentum entropic sums saturate the corresponding entropic uncertainty relations. The latter is also known for the corresponding uncertainty relations of high dimensional harmonic systems [61], which are subject to a quadratic potential. It remains open whether this phenomenon is also fulfilled for high dimensional states of quantum systems moving in general central potentials or, at least, in power-law central potentials.

## Data Availability

Not applicable.
